# A borderline range for Quantiferon Gold In-Tube results

**DOI:** 10.1371/journal.pone.0187313

**Published:** 2017-11-02

**Authors:** Jerker Jonsson, Anna Westman, Judith Bruchfeld, Erik Sturegård, Hans Gaines, Thomas Schön

**Affiliations:** 1 The Public Health Agency of Sweden, Solna, Sweden; 2 Unit of Infectious Diseases, Department of Medicine, Karolinska Institutet, Stockholm, Sweden; 3 Department of Infectious Diseases, Danderyd Hospital, Stockholm, Sweden; 4 Division of Clinical Microbiology, Department of Laboratory Medicine, Karolinska University Hospital Laboratory, Stockholm, Sweden; 5 Department of Infectious Diseases, Karolinska University Hospital Solna, Stockholm, Sweden; 6 Infectious Diseases Research Unit, Department of Translational Medicine, Lund University, Lund, Sweden; 7 Department of Clinical Microbiology and Infectious Diseases, Kalmar County Hospital, Kalmar, Sweden; 8 Division of Medical Microbiology, Department of Clinical and Experimental research, Linköping University, Linköping, Sweden; University of Texas Health Northeast, UNITED STATES

## Abstract

**Objective:**

Interferon gamma release assays like Quantiferon Gold In-Tube (QFT) are used to identify individuals infected with *Mycobacterium tuberculosis*. A dichotomous cut-off (0.35 IU/ml) defines a positive QFT without considering test variability. Our objective was to evaluate the introduction of a borderline range under routine conditions.

**Methods:**

Results of routine QFT samples from Sweden (2009–2014) were collected. A borderline range (0.20–0.99 IU/ml) was introduced in 2010 recommending a follow-up sample. The association between borderline results and incident active TB within 3 to 24 months was investigated through linkage with the national TB-register.

**Results:**

Using the recommended QFT cut-off, 75.1% tests were negative, 21.4% positive and 3.5% indeterminate. In total, 9% (3656/40773) were within the borderline range. In follow-up samples, individuals with initial results between 0.20–0.34 IU/ml and 0.35–0.99 IU/ml displayed negative results below the borderline range (<0.20 IU/ml) in 66.1% (230/348) and 42.5% (285/671) respectively, and none developed incident TB. Among 6712 individuals with a positive initial test >0.99 IU/ml, 65 (0.97%) developed incident TB within 3–24 months.

**Conclusions:**

We recommend retesting of subjects with QFT results in the range 0.20–0.99 IU/ml to enhance reliability and validity of the test. Half of the subjects in the borderline range will be negative at a level <0.20 IU/ml when retested and have a very low risk of developing incident active TB.

## Introduction

Interferon gamma release assays (IGRAs) like Quantiferon Gold In-Tube (QFT) and T-Spot.TB are widely used in low-endemic areas to identify individuals infected by *Mycobacterium tuberculosis*. IGRAs detect memory T-cell responses following previous exposure to *M*. *tuberculosis* antigens and therefore a positive test is not necessarily associated to the presence of viable bacteria [1]. Of note, current tests have a low positive predictive value (PPV) of around 2% for progression into active tuberculosis (TB) within two years according to systematic reviews [2, 3].

In 2010, a borderline range for QFT testing was introduced in Sweden [4] and in the same year, CDC recommended that the role of a borderline range to improve diagnostic accuracy for QFT should be further explored [5]. The selected borderline range was based on retrospective evaluation of 6300 consecutive QFT results assuming an overlap between positive and negative results around the test's recommended cut-off level. The chosen range of 0.2–0.99 comprised ±5% from the cut-off for the 6300 evaluated results and was selected as reasonable for recommendations for testing of a follow-up sample. The same borderline range has later been independently suggested from the QFT result distribution of a recent North American study of health care workers [6, 7]. In addition, several other studies have later suggested borderline ranges typically spanning from 0.2–0.25 to 0.7–0.99 IU/ml [6, 8–15].

Both IGRAs show variability and this has fuelled a debate about serial testing in health care workers [6, 7, 11, 13, 16, 17]. There are several pre-analytical, technical and patient related factors which could influence variability of the QFT-results such as varying blood volumes collected, intense or insufficient shaking and delayed incubation of the tubes at 37°C [12, 15, 18]. A recent systematic review showed a variability of ±0.26–0.7 IU/ml for QFT results in the 0.25–0.8 IU/ml range independent of pre-test probability (indication for testing) [15]. A particularly problematic area are conversions (from negative to positive) or reversions (from positive to negative) around cut-off (≥0.35 IU/ml). In several studies, mainly in health care workers, unexpectedly high reversion rates (20–80%) have been observed [6, 11, 13, 19]. There are considerable doubts whether such reversions represent true immunological events and many authors therefore suggest that short-term reversions are mainly due to test variability [6, 15].

The introduction of a borderline range has not been extensively evaluated under routine conditions. Our aim was to investigate if an introduction of a borderline range (0.2–0.99 IU/ml) with a recommendation for testing a follow-up sample could improve interpretation of QFT-results.

## Methods

### Study population and definitions

The main indications for QFT-testing in Sweden are latent TB infection (LTBI) screening of asylum seekers from high endemic TB countries, planned immunosuppression (e.g. prior to anti-TNF-alfa treatment) and contact investigation [4]. In addition, although not recommended, QFT is sometimes included in the diagnostic work-up of active TB. Sweden is a low-TB incidence country and had an overall incidence of 6.9–7.1/100 000 during the study period (2009–2014). Close to 90% of TB-cases diagnosed in Sweden are of foreign origin [20].

The included QFT test results were from four clinical microbiology laboratories in Sweden covering more than 60% of the Swedish population. All clinical samples analysed for QFT in these four laboratories during the period 1^st^ of January 2009 until 31th of December 2014 were included in the study. Duplicates and samples without a unique national personal identifier (mainly recent immigrants) or with unknown age were excluded from the analysis.

From 2010, a recommendation to request a follow-up sample in a defined borderline range of 0.2–0.99 IU/ml was given in laboratory reports from participating laboratories and this recommendation was also introduced from 2012 in the Swedish national guidelines [4]. In addition to retesting QFT samples with borderline results, an additional sample was requested 8–12 weeks after last possible exposure in contact tracing of individuals with recent TB exposure [4].

In order to facilitate comparisons with the cut-off for a positive test from the manufacturer (0.35 IU/ml), the data is presented as negative (<0.20 IU/ml), borderline negative (0.20–0.34 IU/ml), borderline positive (0.35–0.99 IU/ml) and positive (≥1.0 IU/ml). Reversions are defined as initial positive (≥0.35 IU/ml) tests reverting to negative upon retesting a new sample and conversions are negative results (<0.35 IU/ml) converting to positive. All proportions include indeterminate results in the denominator. Indeterminate results are defined as a test where the negative control was ≥8 IU/ml or the positive control ≤0.5 IU/ml according to recommendations by the manufacturer.

### Linking QFT-results to the Swedish national tuberculosis register

All included samples from individuals with a unique personal identification number were compared with the national TB-register to investigate the association of QFT-results to a diagnosis of active TB. The date of TB-diagnosis was compared to date of QFT-sampling. We included data from the TB-register from 2009-01-01 to 2016-12-31 to link a TB-diagnosis to the QFT-result with a follow-up period of two years or more for all samples. In accordance with previous studies [21], we defined incident TB as active TB occurring from 3 months up to two years after the QFT sample was obtained.

### Quantiferon Gold In-Tube

Quantiferon Gold In-Tube was performed in all laboratories according to the manufacturer`s instructions (Qiagen). All included laboratories provided written guidelines for testing but no further standardization was possible due to the retrospective nature of the data. At the time of sample collection and prior to incubation, the recommendation from all laboratories was to mix the samples thoroughly by shaking the tube 10 times (5 s) to ensure that the entire inner surface of the antigen coated tube was covered with blood.

### Ethical considerations

The project was approved by the Regional Ethical committee in Stockholm (DNR 2014/217-31/4 and 2015/1772-32). The ethical committee waived the need for consent from the participants providing medical samples, as the study was retrospective and the data was de-identified after the comparison with the TB-register and before analysis.

### Statistical analysis

Parametric data are presented as medians and inter-quartile ranges (IQR). Comparisons between groups were performed by chi-square test with Yates correction. A p-value <0.05 was regarded as statistically significant.

## Results

**Fig 1:**
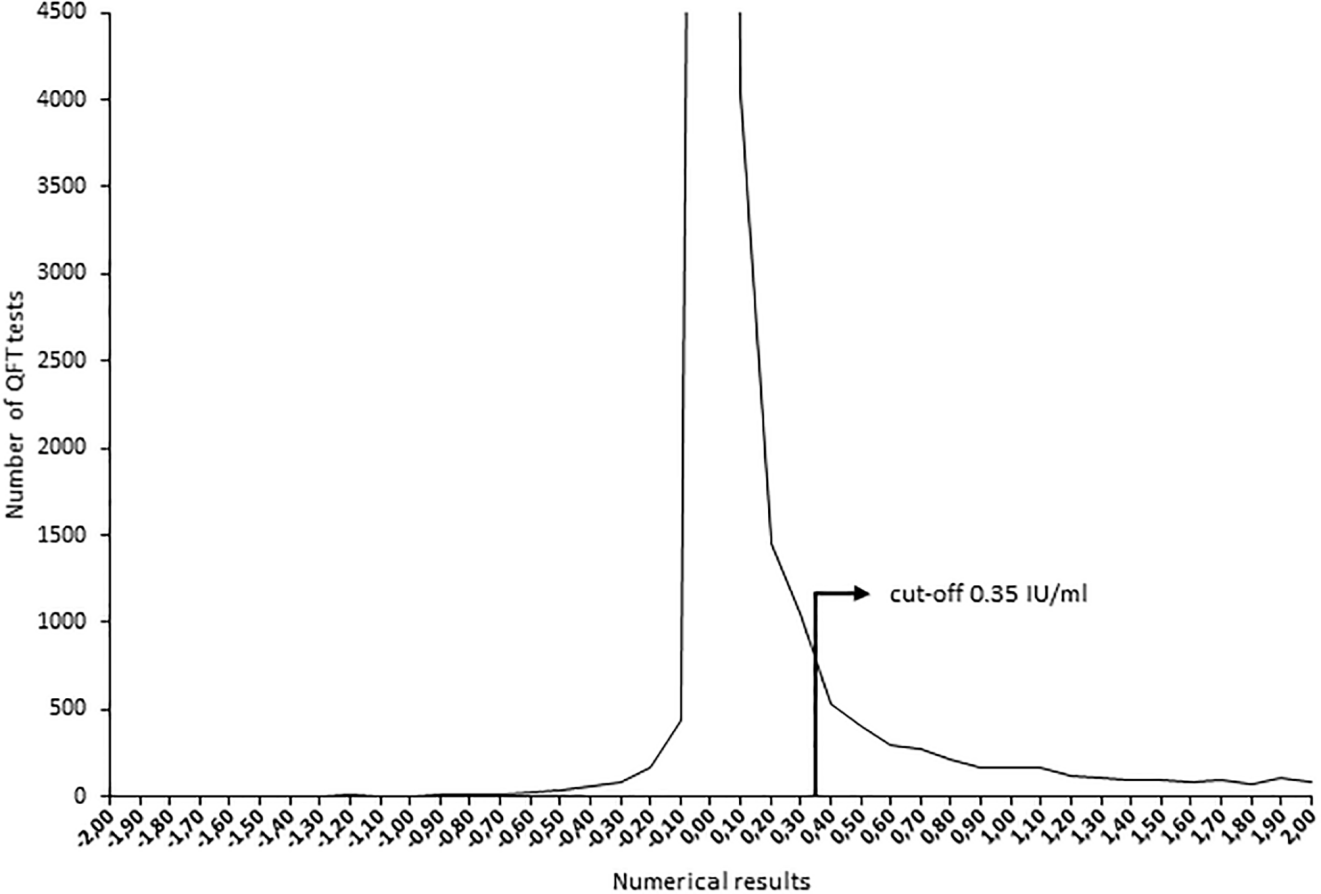
Distribution of numerical QFT results from -2 to +2 IU/ml (1 n = 33225) where the range -0.05 to 0.04 IU/ml reaches outside the graph as the large number of observations in this range distorts the scale (n = 22645).

A total of 48000 QFT test results were collected of which results from 40773 individuals remained after exclusion. Using the recommended QFT cut-off, 75.1% were negative, 21.4% positive and 3.5% indeterminate. There was no clear separation in the distribution of results below and above the cut-off of 0.35 IU/ml, demonstrating an overlap between negative and positive results, (Fig 1). In total, 9% (3656/40773) of tests were within the 0.20–0.99 IU/ml range.

### Follow-up samples from individuals with initial QFT levels between 0.20–0.99 IU/ml

Follow-up samples of individuals with initial QFT results between 0.20–0.34 IU/ml and 0.35–0.99 IU/ml displayed negative results <0.20 IU/ml in 66.1% (230/348) and 42.5% (285/671), respectively (Table 1).

**Table 1:** Categorical distribution of follow-up QFT results when retesting those with initial result in the borderline range (0.20–0.99 IU/ml).

				Result of follow-up QFT test (IU/ml)					
Initial result (IU/ml)	Total (n)	Percent-age retested (n)	Median days to retest (IQR)	Indeter-minate	Negative (<0.20)	Border-line negative (0.2–0.34)	Border-line positive (0.35–0.99)	Positive (>0.99)	Total retested
**Borderline negative (0.20–0.34)**	1664	20.9% (348)	52 (25–112)	1.2% (4)	66.1% (230)	13.2% (46)	12.9% (45)	6.6% (23)	100.0% (348)
**Borderline positive (0.35–0.99)**	1992	33.7% (671)	38 (20–84)	1.3% (9)	42.5% (285)	12.2% (82)	26.7% (179)	17.3% (116)	100.0% (671)
**All borderline (0.20–0.99)**	3656	27.9% (1019)	42 (21–92)	1.3% (13)	50.5% (515)	12.6% (128)	22.0% (224)	13.6% (139)	100.0% (1019)

The results of retesting in borderline range is divided in borderline negative (0.20–0.34 IU/ml) and borderline positive (0.35–0.99 IU/ml) initial result.

In total, 27.9% of borderline reactive samples were retested (1019/3656) within a median of 42 days. Of those samples, 50.5% (515/1019) turned negative <0.20 IU/ml.

In total, 50.5% (515/1019) with an initial QFT level of 0.20–0.99 IU/ml were negative below the borderline range (<0.20 IU/ml, Table 2; S1–S2 Figs) upon retesting.

**Table 2:** Number of cases of active TB (n = 710) per QFT result and time of diagnosis in relation to time of the QFT test.

QFT result by category	Total number tested	Co-prevalent TB (0–3 months)	Incident TB (3–24 months)	TB after 24 months	Total (% of QFT category)
**Indeterminate**	1429	23	4	2	29 (2.0%)
**Negative (<0.2 IU/ml)**	28976	57	14	6	77 (0.3%)
**Borderline negative (0.2–0.34 IU/ml)**	1664	16	2	1	19 (1.1%)
**Borderline positive (0.35–0.99 IU/ml)**	1992	55	11	4	70 (3.5%)
**Positive (>0.99 IU/ml)**	6712	431	65	19	515 (7.7%)
**Total number of cases**	40773	582	96	32	710 (2.2%)

Out of all 96 cases of incident active TB, 13 were found in patients with initial results in the borderline range (0.20–0.99 IU/ml).

**Fig 2:**
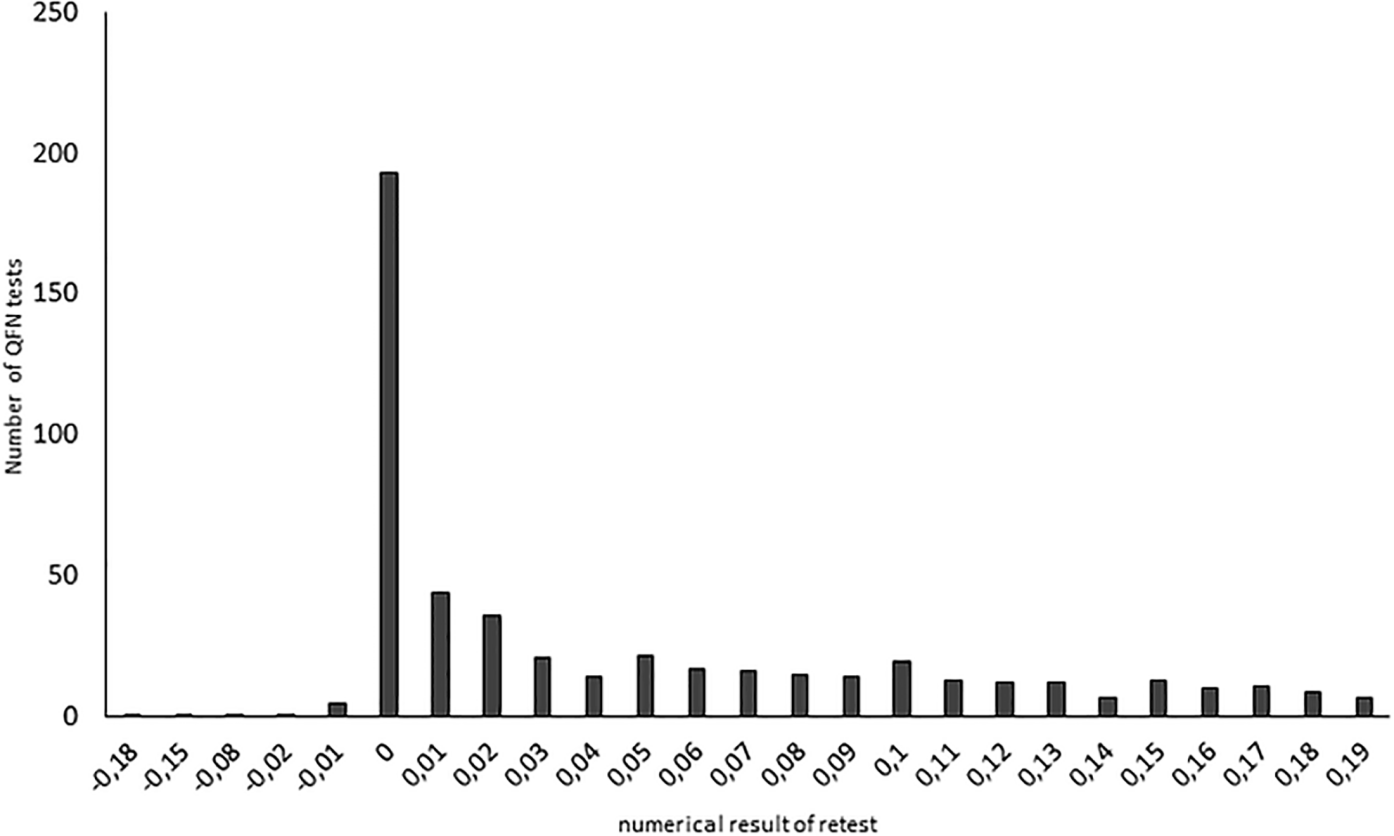
Distribution of numerical results of negative (<0.20 IU/ml) results from a follow-up sample when the initial test were in the borderline range (0.2–0.99 IU/ml, n = 515).

To minimize the risk of new TB exposure affecting the results, we performed a sub-group analysis in individuals with an initial result in the range of 0.20–0.99 IU/ml where a follow-up sample was obtained within four weeks. In this subgroup (n = 459), the proportion of negative results <0.2 IU/ml ranged from 73% (initial test result of 0.20–0.24 IU/ml) and gradually decreased to 29% (initial test result of 0.78–0.99 IU/ml; S1 Fig and S1 Table). The majority of samples in the range 0.2–0.99 IU/ml showing a negative result <0.20 IU/ml (61%) upon retesting, were negative at levels even below 0.05 IU/ml (n = 515; Fig 2).

### Conversions and reversions in follow-up QFT samples initially testing 0.2–0.99 IU/ml

In total, there were 54.7% (367/671) reversions, according to the recommended cut-off (0.35 IU/ml) in follow-up samples from individuals with an initial QFT test in the range 0.35–0.99 IU/ml. Among subjects with an initial QFT level in the range 0.2–0.34 IU/ml, 19.5% (68/348) converted to >0.35 IU/ml. In the subgroup of patients with a follow-up sample within four weeks, there were 59.4% (155/261) reversions and 15.2% (16/105) conversions.

### Association between initial QFT results in the range of 0.2–0.99 IU/ml and development of active TB

During the study period (2009–2014) and until January 2017, 710 cases of active TB had been reported among the patients included in our study (Table 2). The total follow-up time for development of active TB after the initial QFT sample was 175 998 person-years with a median of 4.32 years (2.01–7.99). In total, 96 patients developed active TB 3–24 months after being tested (Table 2). There were significantly more patients who developed incident active TB among those positive >0.99 IU/ml (0.97%; 65/6712) compared to subjects with test results in the borderline range 0.20–0.99 IU/ml (0.56%; 13/3656, p = 0.0008). Among patients with initial tests 0.20–0.99 IU/ml where a second sample was obtained, 0.20% (2/1019) developed incident active TB. Both patients had an initial QFT result of 0.66 IU/ml and 0.89–0.98 IU/ml respectively in the follow-up test. In patients with borderline positive results 0.35–0.99 IU/ml, reversions were found in 54.7% (367/671) and no case of incident TB was found during follow-up among those who reverted.

## Discussion

The present study focused on analysing the introduction of a borderline range around the dichotomous cut-off for QFT to improve clinical interpretation and validity of the test. We show that more than 50% of subjects with QFT results in the borderline range were negative <0.20 IU/ml in follow-up samples and no case of incident TB was found in this subgroup. Based on the results from this large cohort, we recommend the introduction of a borderline range (0.20–0.99 IU/ml), such as has been done for the other IGRA-test T-spotTB [9], in order to improve clinical decision-making.

Limits for a borderline range for QFT has been extensively discussed [6, 8–15]. Additionally, the role of a borderline range to improve accuracy for QFT was identified as an important area for further research by the CDC already in 2010 [5] but only very limited data from routine conditions except for studies on health care workers (HCW) are available. There are several technical factors which could influence the QFT-results as outlined by others and test variability leads to clinical interpretation problems when using a dichotomous cut-off [9, 12, 15]. Our study extend previous findings on the importance of QFT borderline ranges to routine indications for QFT testing and also include analysis of risk of TB activation.

The importance of the variability in QFT testing has become most obvious in the case of serial testing of HCWs in the USA where it coincides with a low PPV in a low-TB risk population [6, 8, 9]. A recent study showed a 77% reversion rate in 1094 HCWs testing QFT positive at less than 1.16 IU/ml [19]. Additionally, a recent systematic review revealed reversions in 44.4% of 818 subjects with repeat testing within 4 weeks.

We show that more than 50% of subjects with QFT results in the borderline range were negative <0.20 IU/ml in follow-up samples obtained within 4 weeks. This strongly indicates a false positive initial result on the basis of the test variability for results ≥0.35–0.99 IU/ml rather than a reversion from an immunological perspective. True reversions may theoretically exist in rare cases where the cell mediated response to IFN-gamma production to *M*. *tuberculosis* antigen is subsiding [9, 12, 15]. Retesting of individuals with results of 0.35–0.99 IU/ml will thus give a more solid basis for clinical interpretation and in particular avoid unnecessary treatment of LTBI when the indication for testing is not clear. The most important indications for retesting a new sample from initially QFT borderline negative subjects (0.20–0.34 IU/ml) are confirmed or suspected recent TB exposure or when immunosuppressive therapy is planned as test variability may lead to a false negative results if a dichotomous cut-off is used.

A novel version of the QFT test (QuantiFERON-TB Gold Plus) was recently launched in Europe and has now also been FDA approved in the USA. Clinical performance data is very limited as pointed out in recently updated CDC guidelines [9, 22–24]. According to the manufacturer, the novel version of the test is improved in terms of sensitivity due to inclusion of a second tube for TB-antigens (TB2) reported to react with CD8+ T-cells (22–24). Direct comparisons to the previous version for the other tube of TB-antigens (TB1) is unfortunately lost due to the omission of the TB 7.7 antigen. Not surprisingly, recent data suggest that the new QFT version will also show a considerable variability around the cut-off at least in the range of the QFT Gold In tube [9, 25].

Our study has several limitations. First, we have no information on the indication for testing nor if treatment was given for LTBI. Treatment for LTBI would reduce the number of cases of incident TB. Second, only about a third of all subjects within the borderline range were retested with a new QFT sample. Third, the association between QFT-test results and development of active TB should be interpreted with caution due to the retrospective design of the study and the small number of patients in the borderline group progressing to active TB.

To the best of our knowledge, this is the largest study investigating the implementation of a borderline range for QFT testing (0.2–0.99 IU/ml) under routine conditions. Based on our results, we recommend an introduction of a borderline QFT range 0.20–0.99 IU/ml in order to improve reliable diagnosis of LTBI and enhance the validity of the test.

## Supporting information

S1 Fig Categorized QFT results (IU/ml) for follow up samples tested within 4 weeks based on the numerical QFT result of the first test (n = 360; indeterminate retest result not shown (n = 6)). (TIF)

S2 Fig Categorized QFT results for follow up samples retested after 4 weeks based on the numerical QFT result of the first test (n = 646; indeterminate retest result not shown (n = 7)). (TIF)

S1 File Data file. Data for uploadQFT171005.
(XLSX)

S1 Table QFT results for initial and follow up samples separated for subjects retested within or beyond 4 weeks. (DOCX)
