# Supplementary figures and images for: A borderline range for Quantiferon Gold In-Tube results
Source: PLoS One. 2017 Nov 2;12(11):e0187313. doi: 10.1371/journal.pone.0187313 (PMC5667766; doi:10.1371/journal.pone.0187313)

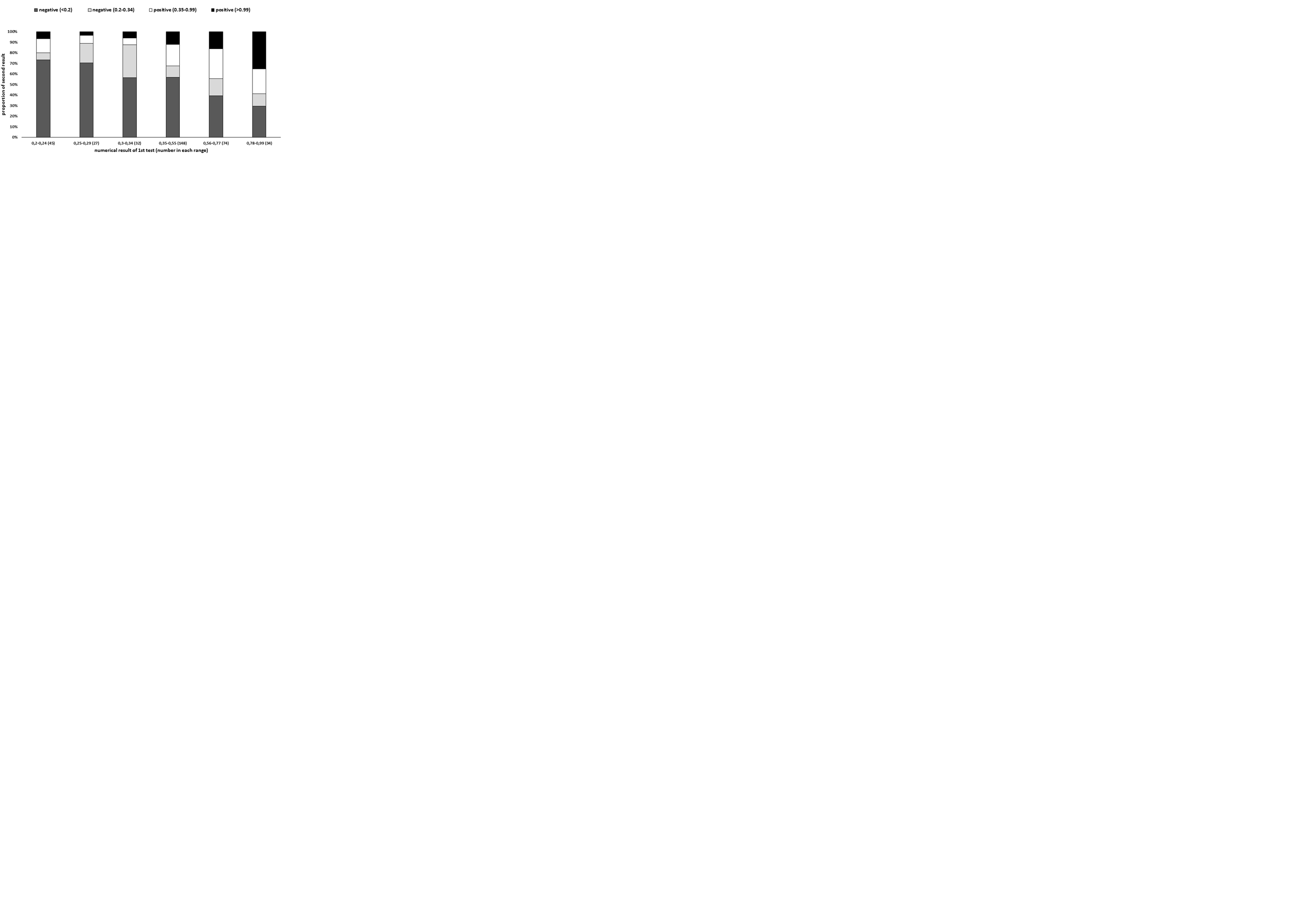

Supplement: S1 Fig — (TIF) [file pone.0187313.s001.tif]

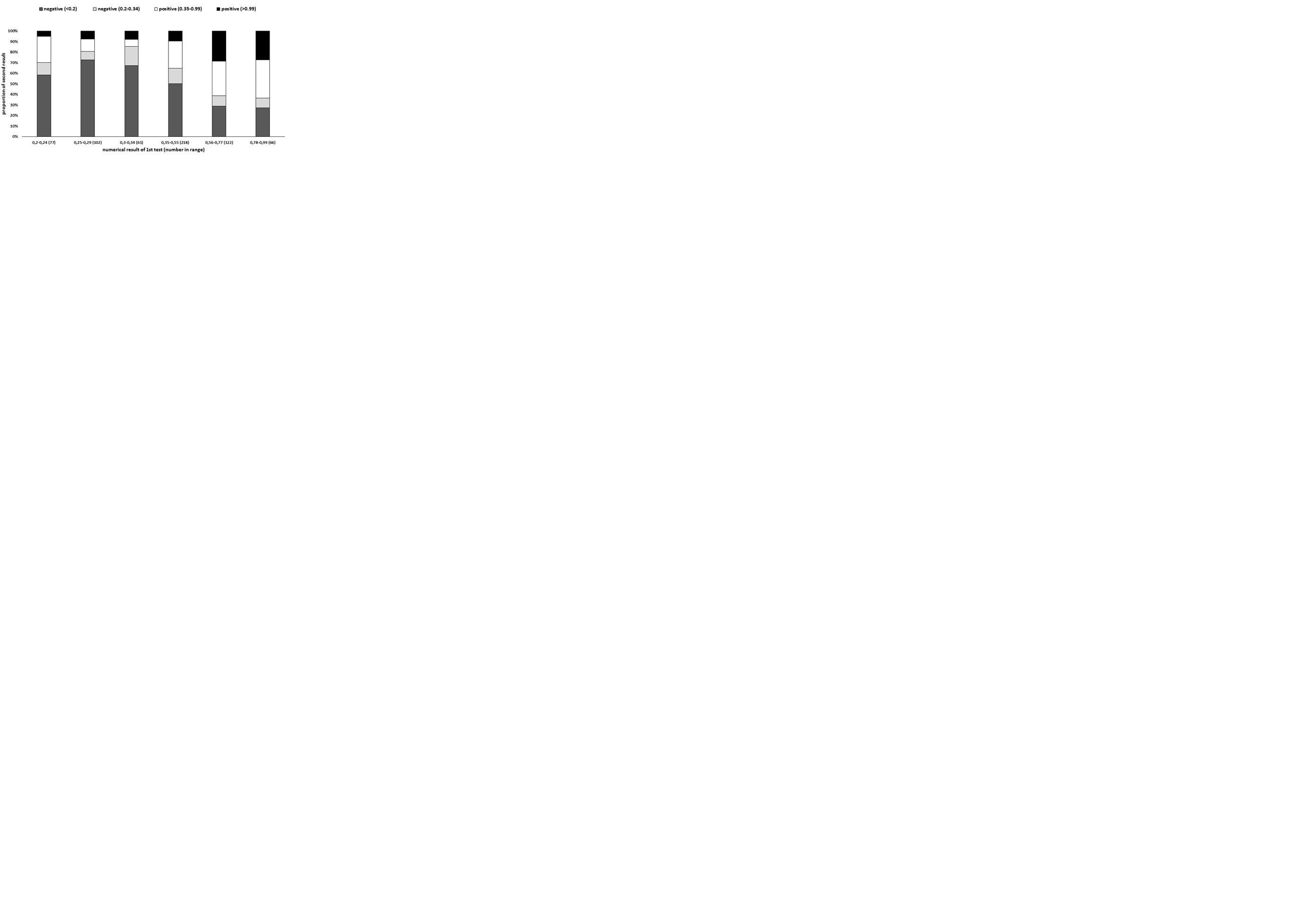

Supplement: S2 Fig — (TIF) [file pone.0187313.s002.tif]
