# Supplementary material for: A borderline range for Quantiferon Gold In-Tube results
Source: PLoS One. 2017 Nov 2;12(11):e0187313. doi: 10.1371/journal.pone.0187313 (PMC5667766; doi:10.1371/journal.pone.0187313)
Supplement: S1 Table — (DOCX) [file pone.0187313.s004.docx]

**S1 Table**. QFT results for initial and follow up samples separated for subjects retested within or beyond 4 weeks.

|  |  |  |  |  |  |  |
| --- | --- | --- | --- | --- | --- | --- |
| **Initial test result (IU/ml)** | **Follow-up result for those retested within 4 weeks** | | | | |  |
|  | Indeterminate | Negative (<0.2) | Negative (0.2-0.34) | Positive (0.35-0.99) | Positive (>0.99) | Total |
| Negative (0.2-0.34) | 1% (1) | 66.7% (70) | 17.1% (18) | 9.5% (10) | 5.7% (6) | 105 |
| Positive (0.35-0.99) | 1.9% (5) | 47.1% (123) | 12.3% (32) | 22.6% (59) | 16.1% (42) | 261 |
| Total | 6 | 193 | 50 | 69 | 48 | 366 |
| **Initial test result (IU/ml)** | **Follow-up result for those retested after more than 4 weeks** | | | | |  |
|  | Indeterminate | Negative (<0.2) | Negative (0.2-0.34) | Positive (0.35-0.99) | Positive (>0.99) | Total |
| Negative (0.2-0.34) | 1.2% (3) | 65.9% (160) | 11.5% (28) | 14.4% (35) | 7.0% (17) | 243 |
| Positive (0.35-0.99) | 1.0% (4) | 39.5% (162) | 12.2% (50) | 29.3% (120) | 18.0% (74) | 410 |
| Total | 7 | 322 | 78 | 155 | 91 | 653 |
